# A Prospective Observational Study on the Evaluation of the Galactomannan Test in the Diagnosis of Invasive Aspergillosis

**DOI:** 10.7759/cureus.95230

**Published:** 2025-10-23

**Authors:** Pankhuri Kumari, Teena Wadhwa, Ashok Kumar, Manish Gaba, Bornali Datta

**Affiliations:** 1 Microbiology, Amrita Hospital Faridabad, Faridabad, IND; 2 Microbiology, Medanta, The Medicity, Gurgaon, IND; 3 Internal Medicine, Max Smart Super Specialty Hospital, New Delhi, IND; 4 Respiratory and Sleep Medicine, Medanta, The Medicity, Gurgaon, IND

**Keywords:** bal, fungal diagnostics, galactomannan assay, invasive aspergillosis, serum gm

## Abstract

Background: Invasive aspergillosis (IA) is a life-threatening opportunistic fungal infection, particularly in immunocompromised patients. Early, reliable diagnosis is essential for timely management.

Objective: To assess the diagnostic performance of the galactomannan enzyme immunoassay (GEIA) in bronchoalveolar lavage (BAL) fluid and serum, comparing with potassium hydroxide (KOH) microscopy, high-resolution CT (HRCT) chest, and culture (as reference).

Methods: In this prospective, single-center observational study (January-December 2018), BAL and/or serum samples from patients clinically suspected of IA were tested using the Platelia™ Aspergillus Galactomannan EIA kit (Bio-Rad, France). Results at two optical density (OD) cut‑offs (≥ 0.5 and > 1.0) were compared against culture, KOH microscopy, and HRCT. Sensitivity, specificity, positive predictive value (PPV), negative predictive value (NPV), accuracy, and 95% confidence intervals were calculated. Pairwise comparisons used McNemar’s test.

Results: Among 159 BAL samples, GEIA positivity was 51.6% (OD ≥ 0.5) and 39.0% (OD > 1.0). At OD ≥ 0.5, BAL galactomannan (GM) sensitivity was 93.3% (95% CI: 83.8-97.9), specificity 71.4% (95% CI: 61.4-79.9), PPV 63.4%, NPV 97.4%, and accuracy 79.9%. At OD > 1.0, sensitivity was 92.6% (95% CI: 82.9-97.9), specificity 88.6% (95% CI: 80.8-93.8), PPV 80.7%, NPV 95.9%, and accuracy 89.9%. KOH microscopy and HRCT in the BAL cohort achieved sensitivities of 90.7% and 85.2% and specificities of 77.1% and 80.0%, respectively. McNemar’s test showed BAL GM (OD > 1.0) was significantly superior to KOH (p = 0.003) and HRCT (p = 0.005). In 60 patients tested with serum GM (OD ≥ 0.5), sensitivity was 84.0% (95% CI: 62.9-95.0), specificity 80.0% (95% CI: 61.4-92.3), PPV 75.0%, NPV 87.5%, and accuracy 81.7%.

Conclusions: In this cohort, BAL GM testing provided excellent sensitivity and, especially at OD > 1.0, improved specificity relative to conventional methods. Serum GM performed moderately well, but with lower sensitivity and specificity. BAL GM (OD > 1.0) may provide an optimal balance for non-culture-based diagnosis of IA, though results should be interpreted in the clinical context. Future multicenter studies incorporating polymerase chain reaction (PCR) and β-D-glucan are warranted to validate these findings.

## Introduction

Invasive aspergillosis (IA) is a severe fungal infection that predominantly affects immunocompromised patients, such as those with prolonged neutropenia, hematological malignancies, or recipients of bone marrow or solid organ transplants [1]. Because delay in diagnosis is associated with poor outcomes, early and accurate detection is essential. High-resolution CT (HRCT) is widely used in suspected pulmonary IA and can show features like the “halo” and “air crescent” signs; however, these are not pathognomonic and often overlap with other pulmonary diseases [2].

While polymerase chain reaction (PCR) detection of *Aspergillus* DNA in bronchoalveolar lavage (BAL) fluid or serum offers high sensitivity, its availability is limited to specialized laboratories and is not routine in many settings [3]. Among newer approaches, the detection of galactomannan (GM), a polysaccharide component of the *Aspergillus* cell wall, by galactomannan enzyme immunoassay (GEIA) has become a key non-culture-based diagnostic modality [4,5]. GM is released during active hyphal growth, and assays are available for BAL, serum, and other body fluids.

Numerous studies suggest GM detection is more sensitive in BAL fluid than in serum [5,6]. Accordingly, the European Organization for Research and Treatment of Cancer/Mycoses Study Group (EORTC/MSG) criteria now include GM assays as part of the diagnostic definitions for IA [7]. Other non-culture methods, such as 1,3-β-D-glucan, have broader fungal specificity and lack specificity for *Aspergillus* species [8].

Given the high mortality associated with IA, there is an ongoing need to refine noninvasive, rapid, and reliable diagnostic methods [9]. This study aims to evaluate the diagnostic performance of GEIA in both BAL and serum, compare it with conventional methods (potassium hydroxide (KOH) microscopy, HRCT, and culture), and assess the impact of different optical density (OD) thresholds in a real-world clinical population.

## Materials and methods

Study design and setting

This was a prospective, single-center observational study conducted in the Pulmonary and Infectious Diseases units of Medanta, The Medicity, Gurgaon, India, from January 2018 to December 2018. The protocol was approved by the institutional ethics committee (DCGI Registration Number: ECR/282/Inst/HR/2013/RR; Reference MICR‑826/2017). Informed consent was obtained from all participants or their legal representatives.

Patient recruitment

We screened 220 consecutive inpatients with clinical suspicion of pulmonary IA, based on fever, cough, hemoptysis, or radiologic pulmonary infiltrates not attributable to other causes. Exclusion criteria included administration in the prior 48 hours of antibiotics known to cause false-positive GM (piperacillin-tazobactam, amoxicillin-clavulanate, ampicillin-sulbactam); receipt of mold-active antifungal therapy (e.g., amphotericin B, itraconazole, voriconazole, caspofungin) before sampling.

Twelve patients were excluded due to recent antibiotic exposure. One patient was lost to follow-up. The final cohort comprised 219 patients. BAL GM was performed in 159 (based on clinical indication for bronchoscopy), and serum GM in 60 patients (often when bronchoscopy was contraindicated or declined). This allocation was pragmatic and potentially introduces selection bias, as acknowledged below.

Sample collection and processing

Galactomannan Testing

BAL and serum samples were processed using the Platelia™ Aspergillus Galactomannan EIA kit (Bio-Rad, France), following the manufacturer’s instructions with slight adaptations. Preprocessing: For each sample, 300 µL was mixed with 100 µL of treatment solution, vortexed, then heated at 120°C for six minutes in a dry heat block. Centrifugation: Samples were centrifuged at 10,000 g for 10 minutes. Assay: Around 50 µL of supernatant was mixed with 50 µL of conjugate in microtiter wells pre-coated with monoclonal antibody EBA-2. Plates were incubated at 37°C for 90 minutes, washed, and then 200 µL of tetramethylbenzidine (TMB) substrate was added for 30 minutes in the dark. The reaction was stopped by adding 100 µL of stopping solution. Reading: Absorbance was read at 450 nm (reference 620 nm). Results were expressed as an OD index relative to internal controls.

We evaluated two cut-off thresholds for positivity: OD ≥ 0.5 (manufacturer’s recommendation) and OD > 1.0 (to improve specificity and reduce false positives). Each run included appropriate positive and negative controls.

Conventional Mycological Methods

KOH mount microscopy: Respiratory specimens (BAL aspirate, bronchial wash) were centrifuged. From the pellet, wet mounts and 10% KOH mounts were prepared, examined under 10× and 40× for fungal hyphae.

Culture: Samples were inoculated on Sabouraud dextrose agar (with and without chloramphenicol (50 mg/L) and cycloheximide (500 mg/L)) and incubated at 25°C and 37°C for up to four weeks. Fungal growth was identified morphologically; isolation of *Aspergillus* spp. was considered confirmatory.

Lactophenol cotton blue (LPCB) staining: Applied to tape lifts from fungal growth to visualize conidial heads, hyphal morphology, and species-level features.

Histopathology: Where feasible, tissue biopsies were examined for evidence of hyphal invasion using standard stains.

Radiology

All patients with suspected pulmonary IA underwent HRCT chest. Radiologic features consistent with IA (halo sign, nodules, wedge-shaped infarcts, cavitation, air-crescent sign) were recorded. Two radiologists independently reviewed scans; discrepancies were resolved by consensus. Inter-rater reliability was assessed using Cohen’s kappa.

Case Classification

Patients were classified per EORTC/MSG 2008 criteria [7] into “proven,” “probable,” “possible,” or “no IA” based on clinical, radiological, and microbiological evidence. For diagnostic performance calculations, only proven and probable cases were considered true positives; possible cases were excluded from test performance metrics.

Statistical analysis

For each diagnostic modality (BAL GM at both cut-offs, serum GM, KOH microscopy, HRCT), sensitivity, specificity, positive predictive value (PPV), negative predictive value (NPV), accuracy, and 95% confidence intervals were calculated using culture (and/or histopathology, when available) as the reference standard. Pairwise comparisons between tests used McNemar’s test for paired proportions. Inter-rater reliability for KOH and HRCT interpretation was calculated using Cohen’s kappa statistic. A p-value < 0.05 was deemed statistically significant. Statistical analyses were performed using IBM SPSS Statistics for Windows, Version 25 (Released 2017; IBM Corp., Armonk, New York, United States).

## Results

Of the 219 patients included, the mean age was 58.6 ± 17.4 years; 61-70 years was the commonest decade. Male patients numbered 145 (66.2%), and females 74 (33.8%). Among 130 patients ultimately classified as proven or probable IA, underlying risk factors included hematological malignancies (26.2%, n = 34), neutropenia (17.1%, n = 22), non-hematological malignancy (13.4%, n = 18), and solid organ transplantation (10.5%, n = 14). Other risk factors (diabetes mellitus, chronic lung disease, corticosteroid therapy) accounted for 34.1% (n = 45) (Table 1).

**Table 1:** Demographic and baseline characteristics (N = 219) Data are presented as Mean ± SD for continuous variables and N (%) for categorical variables. IA: invasive aspergillosis; COPD: chronic obstructive pulmonary disease

Parameter	Value
Mean Age (years)	58.6 ± 17.4
Most Common Age Group	61–70 years
Gender Distribution	
- Male	145 (66.2%)
- Female	74 (33.8%)
Risk Factors in IA-positive Cases	
- Hematological malignancy	34 (26.2%)
- Neutropenia	22 (17.1%)
- Non-hematological malignancy	18 (13.4%)
- Solid organ transplant	14 (10.5%)
- Other (e.g., diabetes, COPD)	45 (34.1%)

Galactomannan enzyme immunoassay in bronchoalveolar lavage samples

Out of 159 patients who underwent BAL sampling, GM positivity at OD ≥ 0.5 was observed in 82 (51.6%) and negativity in 77 (48.4%). Culture was positive in 54 (34.0%) and negative in 105 (66.0%).

OD ≥ 0.5: Among these, 52 were true positives, 75 true negatives, 30 false positives, and two false negatives. Sensitivity = 93.3% (95% CI: 83.8-97.9), specificity = 71.4% (95% CI: 61.4-79.9), PPV = 63.4%, NPV = 97.4%, accuracy = 79.9%.

OD > 1.0: GM positivity was 39.0% (n = 62) and negativity 61.0% (n = 97). The culture positivity distribution was unchanged. Here, true positives = 50, true negatives = 93, false positives = 12, false negatives = 4. Sensitivity = 92.6% (95% CI: 82.9-97.9), specificity = 88.6% (95% CI: 80.8-93.8), PPV = 80.7%, NPV = 95.9%, accuracy = 89.9%.

KOH microscopy in the same BAL cohort had a sensitivity = 90.7%, specificity = 77.1%, PPV = 67.1%, NPV = 94.2%, and accuracy = 81.8%. HRCT showed sensitivity = 85.2%, specificity = 80.0%, PPV = 68.7%, NPV = 91.3%, accuracy = 81.8% (Table 2).

**Table 2:** Diagnostic performance of BAL-based tests compared to KOH mound and HRCT chest (n = 159) Statistical analysis using the Chi-square test BAL: bronchoalveolar lavage; HRCT: high-resolution CT; GM: galactomannan; PPV: positive predictive value; NPV: negative predictive value; KOH: potassium hydroxide

Test	Sensitivity (%)	Specificity (%)	PPV (%)	NPV (%)	Accuracy (%)	χ² Value	p-value
BAL GM (OD > 0.5)	93.3	71.4	63.4	97.4	79.9	72.53	< 0.001
BAL GM (OD > 1.0)	92.6	88.6	80.7	95.9	89.9	91.68	< 0.001
KOH Mount	90.7	77.1	67.1	94.2	81.8	74.21	< 0.001
HRCT Chest	85.2	80.0	68.7	91.3	81.8	69.82	< 0.001

McNemar’s test comparing BAL GM (OD > 1.0) vs. KOH: p = 0.003; vs. HRCT: p = 0.005, indicating statistically significant superiority. Of the 159 BAL patients, 101 (63.5 %) were classified as proven or probable IA (six proven, 75 probable; possible cases n = 20 excluded from test performance analysis). The remaining 58 (36.5 %) had no evidence of IA per EORTC/MSG criteria.

Galactomannan enzyme immunoassay in serum samples

In 60 patients, serum GM testing was performed using OD ≥ 0.5 as a threshold. Twenty-eight (46.7 %) were GM-positive, 32 (53.3 %) were negative. Culture positivity was seen in 25 (41.7 %) and negative in 35 (58.3 %). Among them, 21 were true positives, 28 true negatives, seven false positives, and four false negatives. Sensitivity = 84.0% (95% CI: 62.9-95.0), specificity = 80.0% (95% CI: 61.4-92.3), PPV = 75.0%, NPV = 87.5%, accuracy = 81.7%. (Table 3)

**Table 3:** Diagnostic performance of serum-based tests compared to culture (n = 60) Statistical analysis by the Chi-square test BAL: bronchoalveolar lavage; HRCT: high-resolution CT; GM: galactomannan; PPV: positive predictive value; NPV: negative predictive value; OD: optical density

Test	Sensitivity (%)	Specificity (%)	PPV (%)	NPV (%)	Accuracy (%)	χ² Value	p-value
Serum GM (OD > 0.5)	84.0	80.0	75.0	87.5	81.7	41.39	<0.001
KOH Mount	80.0	77.1	71.4	84.4	78.3	36.73	<0.001
HRCT Chest	80.0	74.3	69.0	83.9	77.1	34.58	<0.001

In this subgroup, two (3.3 %) were classified as proven IA, 18 (30 %) probable, 10 (16.7 %) possible, and 30 (50 %) no IA classification. Inter-rater reliability for KOH microscopy slides, κ = 0.82; for HRCT readings, κ = 0.79 (both indicating substantial agreement).

## Discussion

In this study, the most common risk factor identified among patients diagnosed with IA was hematological malignancy, with acute myeloproliferative leukemia (AML) being the predominant condition. This finding aligns with previous studies, such as that by Montagna et al. [10], which reported hematological malignancies (48.7%), particularly AML (55.7%), as the leading predisposing factor. Culture positivity for *Aspergillus* spp. was observed in 34% of BAL samples and 41.7% of serum-associated specimens. Given the known moderate sensitivity of culture, these findings underscore the importance of incorporating non-culture-based diagnostic tools such as GM assays. These results demonstrate moderate sensitivity for culture and reinforce the need for non-culture-based diagnostic methods, particularly in high-risk patients. Previous reports have shown variable sensitivity for GM detection, ranging from 0% to 90% depending on methodology and patient populations [11-13]. HRCT findings were positive in 42.1% of patients with BAL GM testing. The observed sensitivity and specificity of HRCT (85.19% and 80%, respectively) are consistent with findings from earlier studies by Kitasato et al. [14] and Kohno et al. [15].

Radiological signs in our cohort were variable. Unlike the findings by Greene et al. [16], where the halo sign was seen in 61% and air crescent in 10% of patients, these features were less common in our patients. Other findings, such as micronodules, consolidation, infarct-shaped nodules, and cavitary lesions, were also observed but varied among patients. This supports the notion that radiologic features alone are insufficient for a definitive diagnosis, especially in immunocompromised individuals. Serum GM testing was evaluated in 60 patients, with HRCT positivity in 33 (55%) of them. The sensitivity and specificity of HRCT in this subgroup were 96% and 74.29%, respectively. This again highlights that HRCT, although valuable, lacks specificity and should be interpreted alongside microbiological or serological findings. Histopathology was performed in 20 patients, with eight showing positive findings. Literature suggests that histopathological examination has a diagnostic accuracy of approximately 78% for identifying *Aspergillus* spp. [17], reinforcing its complementary role in diagnosis.

In our study, BAL GM testing using a cut-off OD index > 0.5 yielded a high sensitivity (93.3%) but lower specificity (71.4%). This correlates with findings by D’Haese et al. [18] and Taghizadeh-Armaki et al. [19], who reported similar diagnostic performance using the same threshold. When a higher cut-off of OD > 1.0 was applied, the specificity increased significantly to 88.6%, while sensitivity remained acceptable at 92.6%. This inverse relationship between sensitivity and specificity with changing cut-off values is consistent with earlier studies by Maertens et al. [20], Husain et al. [21], and Prattes et al. [22].

Maya Hites et al. [23] conducted a study involving 110 patients and evaluated GM in both BAL and serum using a cut-off of OD > 0.5. Their results showed a sensitivity and specificity of 88% and 87% in BAL and 82% and 81% in serum, respectively. Our findings align closely with this, further validating the diagnostic utility of BAL GM testing.

In our cohort, 159 patients were evaluated using BAL GM. At a cut-off OD > 0.5, positivity was observed in 82 patients (51.6%), with a sensitivity of 96.3%, specificity of 71.4%, PPV of 63.4%, and NPV of 97.4%. When the cut-off was increased to OD > 1.0, BAL GM positivity dropped to 39% (n = 62), but specificity improved to 88.6%, with sensitivity still high at 92.6%, PPV at 80.7%, and NPV at 95.9%. These results underscore that while a lower cut-off increases detection, it also increases false positives. Conversely, a higher cut-off offers better specificity without a major loss in sensitivity.

For serum GM testing (n = 60), at a cut-off OD > 0.5, the sensitivity was 84%, specificity 80%, PPV 75%, and NPV 87.5%. These findings are in agreement with previous studies, such as those by Lamoth et al. [24]. Sarrafzadeh et al. [25] also demonstrated that increasing the cut-off to OD > 1.5 improved specificity to 72.2% but reduced sensitivity to 69.2%, highlighting the same trade-off seen in our BAL results.

Overall, the diagnostic accuracy of BAL GM was superior compared to serum GM, KOH microscopy, and HRCT. McNemar’s test demonstrated statistically significant superiority of BAL GM (OD > 1.0) over KOH microscopy (p = 0.003) and HRCT (p = 0.005) in terms of diagnostic accuracy. Among all tests evaluated, BAL GM with a cut-off OD >1.0 provided the best balance of sensitivity and specificity, making it a valuable tool in diagnosing IA, particularly in immunocompromised patients where early diagnosis is critical for improved outcomes.

Limitations

The limitations of this study have been discussed. Single-center design may limit generalizability. The serum GM subgroup was relatively small (n = 60), reducing statistical power. Allocation to BAL or serum testing was based on clinical decision-making, introducing potential selection bias. Use of culture (with limited sensitivity) as a primary reference likely underestimates true disease burden. We did not include PCR assays or 1,3-β-D-glucan testing, which might have further refined diagnostic performance and allowed more comprehensive comparisons. Culture positivity was modest (34% in BAL). Given the known limited sensitivity of culture in invasive fungal disease, we acknowledge that using culture (and histopathology when available) as a “gold standard” is imperfect and may underestimate true disease presence. This limitation may bias estimates of sensitivity and specificity.

Clinical implications

In settings where bronchoscopy is feasible, BAL GM testing using OD > 1.0 offers a practical and high-performing adjunct to conventional diagnostics, potentially enabling earlier initiation of targeted antifungal therapy. Careful clinical correlation remains essential, especially in cases with discordant results. For patients unable to undergo bronchoscopy, serum GM remains useful but with acknowledged performance limitations.

## Conclusions

In our study cohort, BAL GM testing demonstrated excellent sensitivity and, at a higher OD cut-off, markedly improved specificity, outperforming conventional KOH microscopy and HRCT in diagnosing IA. Serum GM showed moderate accuracy but lagged behind BAL performance. An OD threshold of > 1.0 in BAL may strike an optimal balance between sensitivity and specificity in clinical settings. These findings support the integration of BAL GM testing into diagnostic protocols, but validation through larger multicenter studies, including PCR and β-D-glucan assays, is recommended before broad adoption.
